# Development and validation of a diagnostic model for differentiating tuberculous spondylitis from brucellar spondylitis using machine learning: A retrospective cohort study

**DOI:** 10.3389/fsurg.2022.955761

**Published:** 2023-01-06

**Authors:** Parhat Yasin, Muradil Mardan, Tao Xu, Xiaoyu Cai, Yakefu Abulizi, Ting Wang, Weibin Sheng, Mardan Mamat

**Affiliations:** ^1^ Department of Spine Surgery, The First Affiliated Hospital of Xinjiang Medical University, Urumqi, Xinjiang, China; ^2^ School of Medicine, Tongji University, Shanghai, China

**Keywords:** tuberculous spondylitis (TS), brucellar spondylitis (BS), magnetic resonance imaging (MRI), computed tomography (CT), x-ray, machine learning

## Abstract

**Background:**

Tuberculous spondylitis (TS) and brucellar spondylitis (BS) are commonly observed in spinal infectious diseases, which are initially caused by bacteremia. BS is easily misdiagnosed as TS, especially in underdeveloped regions of northwestern China with less sensitive medical equipment. Nevertheless, a rapid and reliable diagnostic tool remains to be developed and a clinical diagnostic model to differentiate TS and BS using machine learning algorithms is of great significance.

**Methods:**

A total of 410 patients were included in this study. Independent factors to predict TS were selected by using the least absolute shrinkage and selection operator (LASSO) regression model, permutation feature importance, and multivariate logistic regression analysis. A TS risk prediction model was developed with six different machine learning algorithms. We used several metrics to evaluate the accuracy, calibration capability, and predictability of these models. The performance of the model with the best predictability was further verified with the area under the curve (AUC) of the receiver operating characteristic (ROC) curve and the calibration curve. The clinical performance of the final model was evaluated by decision curve analysis.

**Results:**

Six variables were incorporated in the final model, namely, pain severity, CRP, x-ray intervertebral disc height loss, x-ray endplate sclerosis, CT vertebral destruction, and MRI paravertebral abscess. The analysis of appraising six models revealed that the logistic regression model developed in the current study outperformed other methods in terms of sensitivity (0.88 ± 0.07) and accuracy (0.79 ± 0.07). The AUC of the logistic regression model predicting TS was 0.86 (95% CI, 0.81–0.90) in the training set and 0.86 (95% CI, 0.78–0.92) in the validation set. The decision curve analysis indicated that the logistic regression model displayed a higher clinical efficiency in the differential diagnosis.

**Conclusions:**

The logistic regression model developed in this study outperformed other methods. The logistic regression model demonstrated by a calculator exerts good discrimination and calibration capability and could be applicable in differentiating TS from BS in primary health care diagnosis.

## Introduction

Tuberculosis (TB) and brucellosis are severe infectious diseases that are threatening human beings. According to the global tuberculosis report (2014), TB remains one of the world's deadliest communicable diseases, and in 2013, approximately 9.0 million people developed TB, among which 1.5 million died from the disease (1), and another recent report showed that 1.6 million people died from TB in 2017 (2). Brucellosis, which is caused by *Brucella melitensis*, is a serious zoonotic disease that causes more than 500,000 human infections worldwide annually (3). Spinal tuberculosis (STB) is not a rare presentation of extrapulmonary tuberculosis. About 1%–2% of all cases of TB are diagnosed as STB, and these patients represent 10%–15% of extrapulmonary TB, of which nearly half involve the musculoskeletal system (4). About 6%–12% of brucellosis cases may suffer a spinal illness, which is the latent reason for the deformities and permanent neurologic deficiencies (5–8). TS and BS are commonly observed in spinal infectious diseases, which are initially caused by bacteremia. They mostly occur in the thoracolumbar segment of the spine. Both TS and BS present several similar clinical performances, such as low-grade fever, including dull pain or discomfort of the dorsum, and elevated inflammatory mediators; hence, distinguishing TS from BS is challenging and BS is commonly misdiagnosed as TS. Currently, the most effective and accurate method for distinguishing TS from BS is based on biopsy and the isolation, culture, and identification of mycobacteria from patient specimens, but it is laborious and time-consuming (9). Hence, developing rapid, cost-effective, and accurate diagnostic methods is urgently desired and of great clinical significance. In this study, we report the development and validation of a machine learning algorithm-based diagnostic model to differentiate betweenthe acute and subacute stages: TS and BS. The predictive model presented in this article follows the TRIPOD Checklist (10).

## Materials and methods

The research was conducted under the approval of the ethics committee of Xinjiang Medical University Affiliated First Hospital, Urumqi, and individual agreements for this retrospective analysis were waived.

### Patients

Patients admitted to the Department of Spine Surgery between January 2018 and December 2021 and considered as spinal TS (*n* = 275, primary cohort: 612) or BS (*n* = 135, primary cohort: 209) (Table 1) were included in this population-based retrospective cohort study with ethical approval of the ethical review committee board of Xinjiang Medical University Affiliated First Hospital. Patients included in this study met the following criteria: (1) diagnosed with spinal tuberculosis or brucellar spondylitis in the acute and subacute stages; (2) accepted surgery therapy; (3) the collected information, especially imaging materials, was complete and available; and (4) age ≥18 years. Patients who met the following exclusion criteria were excluded from analysis: (1) diagnosed with malignant cancer, hematological diseases, and hepatology disease; (2) spine out of alignment; (3) revision spinal surgery; (4) scoliosis deformity; (5) pyogenic spondylitis; (6) spinal hydatid; (7) age <18 years; and (8) patients with missing data were ≥10%.

**Table 1:** Baseline characteristics of patients.

Variables	Total (*N *= 410)	BS (*N *= 135)	TS (*N *= 275)	*p*-Value
Age (years)	51.6 ± 16.1	51.8 ± 12.2	51.4 ± 17.7	0.780
Gender				**<0.001**
Female	166 (40.5%)	36 (26.7%)	130 (47.3%)	
Male	244 (59.5%)	99 (73.3%)	145 (52.7%)	
Ethnicity				0.181
Han	126 (30.7%)	52 (38.5%)	74 (26.9%)	
Kazak	33 (8.05%)	10 (7.41%)	23 (8.36%)	
Mongolian	7 (1.71%)	1 (0.74%)	6 (2.18%)	
Others	23 (5.61%)	6 (4.44%)	17 (6.18%)	
Uygur	221 (53.9%)	66 (48.9%)	155 (56.4%)	
BMI (kg/m^2^)	23.1 ± 3.03	23.61 ± 2.86	22.8 ± 3.09	0.011
Fever				**<0.001**
High	73 (17.8%)	43 (31.9%)	30 (10.9%)	
Low	337 (82.2%)	92 (68.1%)	245 (89.1%)	
Pain severity				**<0.001**
Moderate	192 (46.8%)	41 (30.4%)	151 (54.9%)	
Severe	218 (53.2%)	94 (69.6%)	124 (45.1%)	
History of weight loss				0.483
No	259 (63.2%)	89 (65.9%)	170 (61.8%)	
Yes	151 (36.8%)	46 (34.1%)	105 (38.2%)	
Past history of tuberculosis in other solid organs				0.181
No	324 (79.0%)	101 (74.8%)	223 (81.1%)	
Yes	86 (21.0%)	34 (25.2%)	52 (18.9%)	
WBC (×10^9^/L)	6.56 ± 2.15	6.62 ± 2.10	6.53 ± 2.17	0.667
ESR (mm/h)	45.60 ± 17.61	44.4 ± 15.4	46.1 ± 18.6	0.327
CRP (mg/L)	44.30 ± 37.00	30.9 ± 23.31	50.8 ± 40.5	**<0.001**
Hb (g/L)	126 ± 17.7	130 ± 16.6	124 ± 18.0	**0.004**
TG (mmol/L)	1.25 ± 0.54	1.37 ± 0.62	1.19 ± 0.49	**0.003**
TC (mmol/L)	3.95 ± 0.92	4.15 ± 0.86	3.85 ± 0.93	**0.002**
HDL-C (mmol/L)	1.00 ± 0.31	0.97 ± 0.30	1.02 ± 0.31	0.155
LDL-C (mmol/L)	2.69 ± 0.77	2.79 ± 0.69	2.65 ± 0.81	0.058
ALB (g/L)	37.8 ± 5.71	37.1 ± 6.08	38.1 ± 5.49	0.093
AST (U/L)	24.0 ± 17.5	27.0 ± 17.0	22.5 ± 17.5	0.012
ALT (U/L)	25.9 ± 27.6	34.3 ± 29.0	21.8 ± 26.0	**<0.001**
GGT (U/L)	51.9 ± 45.4	55.8 ± 43.1	50.0 ± 46.5	0.215
ALP (U/L)	103 ± 42.8	108 ± 39.7	101 ± 44.0	0.072
Location				
C	10 (2.44%)	7 (5.19%)	3 (1.09%)	
C + T	2 (0.49%)	1 (0.74%)	1 (0.36%)	
L	233 (56.8%)	90 (66.7%)	143 (52.0%)	
L + S	52 (12.7%)	27 (20.0%)	25 (9.09%)	
S	1 (0.24%)	0 (0.00%)	1 (0.36%)	
T	90 (22.0%)	6 (4.44%)	84 (30.5%)	
T + L	22 (5.37%)	4 (2.96%)	18 (6.55%)	
Segment	2.48 ± 0.96	2.29 ± 0.66	2.57 ± 1.07	**0.001**
MRI spinal stenosis				**0.001**
No	273 (66.6%)	106 (78.5%)	167 (60.7%)	
Yes	137 (33.4%)	29 (21.5%)	108 (39.3%)	
MRI paravertebral abscess				**<0.001**
No	169 (41.2%)	85 (63.0%)	84 (30.5%)	
Yes	241 (58.8%)	50 (37.0%)	191 (69.5%)	
MRI psoas abscess				0.116
No	259 (63.2%)	93 (68.9%)	166 (60.4%)	
Yes	151 (36.8%)	42 (31.1%)	109 (39.6%)	
MRI epidural abscess				0.973
No	320 (78.0%)	106 (78.5%)	214 (77.8%)	
Yes	90 (22.0%)	29 (21.5%)	61 (22.2%)	
CT vertebral destruction				**<0.001**
Mild (≤1/3)	129 (31.5%)	68 (50.4%)	61 (22.2%)	
Severe (>1/3)	281 (68.5%)	67 (49.6%)	214 (77.8%)	
CT marginal osteophytes				0.429
No	186 (45.4%)	57 (42.2%)	129 (46.9%)	
Yes	224 (54.6%)	78 (57.8%)	146 (53.1%)	
CT endplate sclerosis				0.022
No	267 (65.1%)	77 (57.0%)	190 (69.1%)	
Yes	143 (34.9%)	58 (43.0%)	85 (30.9%)	
CT spinal stenosis				**0.005**
No	317 (77.3%)	116 (85.9%)	201 (73.1%)	
Yes	93 (22.7%)	19 (14.1%)	74 (26.9%)	
CT paravertebral abscess				**0.001**
No	198 (48.3%)	81 (60.0%)	117 (42.5%)	
Yes	212 (51.7%)	54 (40.0%)	158 (57.5%)	
CT epidural abscess				0.737
No	366 (89.3%)	122 (90.4%)	244 (88.7%)	
Yes	44 (10.7%)	13 (9.63%)	31 (11.3%)	
X-ray intervertebral disc height loss				**<0.001**
No	166 (40.5%)	84 (62.2%)	82 (29.8%)	
Yes	244 (59.5%)	51 (37.8%)	193 (70.2%)	
X-ray endplate sclerosis				**<0.001**
No	248 (60.5%)	53 (39.3%)	195 (70.9%)	
Yes	162 (39.5%)	82 (60.7%)	80 (29.1%)	
X-ray osteophytes				**<0.001**
No	251 (61.2%)	63 (46.7%)	188 (68.4%)	
Yes	159 (38.8%)	72 (53.3%)	87 (31.6%)	

BMI, body mass index (kg/m^2^); WBC, preoperative white blood cell ( × 10^9/^L); ESR, preoperative erythrocyte sedimentation rate (mm/h); CRP, preoperative C-reactive protein (mg/L); Hb, preoperative hemoglobin (g/L); TG, preoperative total triglyceride (mmol/L); TC, preoperative total cholesterol (mmol/L); HD-C, preoperative high-density lipoprotein cholesterol (mmol/L); LDL-C, preoperative low-density lipoprotein cholesterol (mmol/L); ALB, preoperative operative albumin (g/L); AST, preoperative aspartate aminotransferase (U/L); ALT, preoperative alanine aminotransferase (U/L); GGT, preoperative gamma-glutamyl transferase (U/L); ALP, alkaline phosphataseU/L; C, cervical spine; T, thoracic spine; T + L,thoracolumbar spine; L, lumbar spine; L + S, lumbosacral spine.

The diagnosis, referred to as a response variable in our research, was obtained from symptoms, signs, laboratory tests, and imaging features. TS and BS share similar clinical presentation along with the systemic constitutional manifestation, characterized by sweating, fever, local pain, fatigue, etc. Imaging revealed mild or severe vertebral destruction, intervertebral disc height loss, cold abscess, etc. Laboratory tests included erythrocyte sedimentation rate (ESR), C-reactive protein (CRP), and routine blood tests, which are considered nonspecific. Specific tests comprised positive results of enzyme-linked immunospot assay (T-SPOT.TB), the presence of *Mycobacterium tuberculosis* based on acid-fast bacilli in Ziehl–Neelsen-stained smears, growth in cultures, and/or biopsy examination for TS and *Brucella* agglutination titer test (1:160 or higher) and isolation of *Brucella* species from blood, bone marrow, or other tissues for BS.

### Collection of data

**Figure 1:**
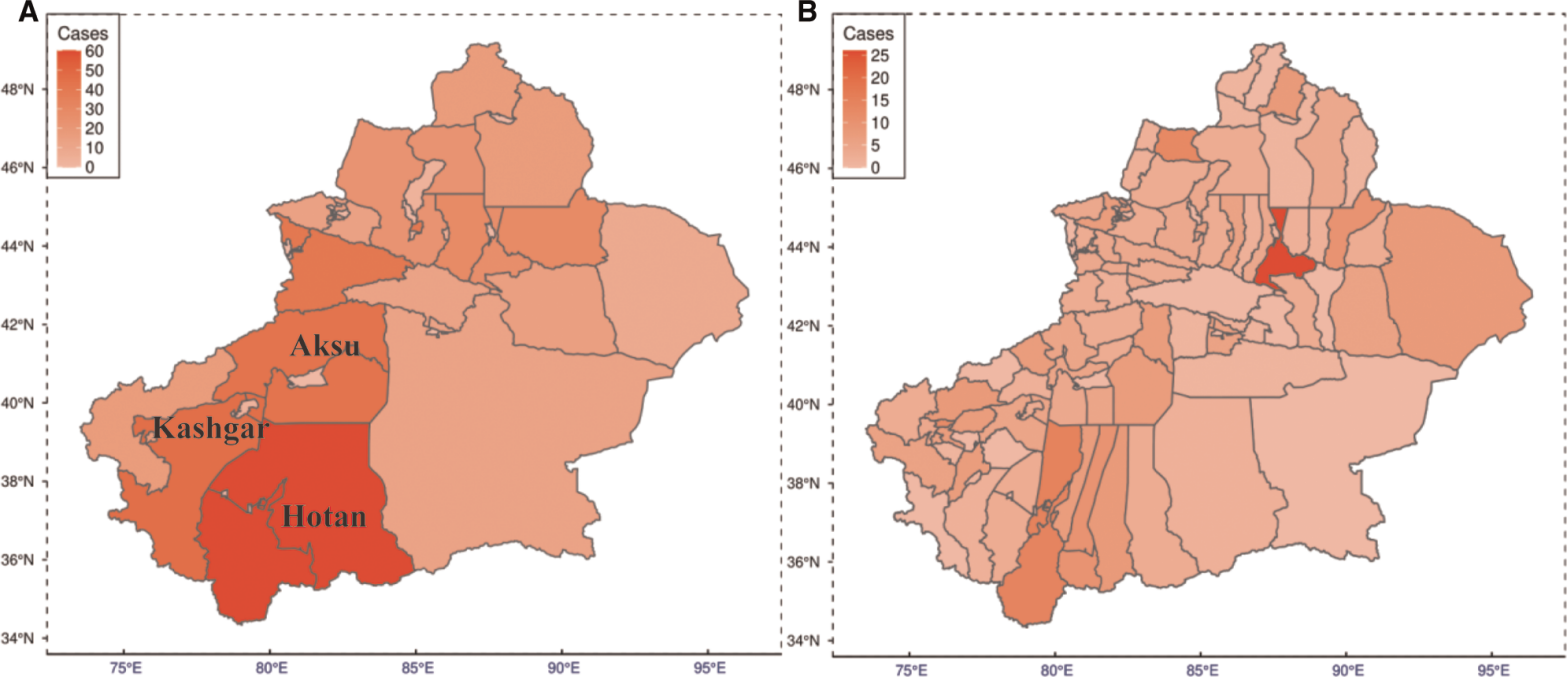
Prevalence of TS and BS among northwestern Chinese residents. (**A**) Prevalence map of the regional level. (**B**) Prevalence map of the county level. TS, tuberculous spondylitis; BS, brucellar spondylitis.

Demographic, clinical, and imaging data were collected for each case, including age, gender, location that can be used to estimate the disease epidemiology characteristic (map source: http://datav.aliyun.com/portal/school/atlas/area_selector) (as is shown in Figure 1), the body mass index (BMI), the level of pain degree divided into two categories based on the visual analog scale (moderate, VAS ≤ 5; severe, VAS > 5), the fever grade measured at the patient’s first visit also divided into two categories (low, <38.5°C; high, ≥38.5°C), preoperative ESR, preoperative CRP, preoperative white blood cell (WBC) count, preoperative hemoglobin, history of weight loss, history of tuberculosis in other solid organs, preoperative low-density lipoprotein cholesterol (LDL-C), preoperative high-density lipoprotein cholesterol (HDL-C), preoperative total cholesterol (TC), preoperative total triglyceride (TG), preoperative albumin (Alb), preoperative gamma-glutamyl transferase (GGT), preoperative alanine aminotransferase (ALT), preoperative aspartate aminotransferase (AST), preoperative alkaline phosphatase (ALP), the level of involvement, the number of affected vertebra, magnetic resonance imaging (MRI) findings including abscess (paravertebral abscess, epidural abscess, psoas abscess) and spinal stenosis, and computed tomography (CT) findings including vertebral destruction, marginal osteophytes, endplate sclerosis, spinal stenosis, paravertebral abscess, and epidural abscess. We defined severe vertebral destruction as one-third or higher vertebral damage. X-ray findings included intervertebral disc height, osteophytes, endplate sclerosis, and bone bridge. All images used in this study were reviewed and analyzed by a chief physician blinded to clinical and laboratory results. We imputed the missing data (<10%) using the MICE package (version 3.14.0) (11).

### Feature selection

We identified candidate predictors through the least absolute shrinkage and selection operator (LASSO) model owing to its attribution of compression estimation algorithms in high-dimensional regression and the importance score of each predictor via the permutation importance approach using the random forest classification model. After applying the LASSO regression model and permutation feature importance method to the training set, respectively, we initially screened variables (12). We chose the top 10 variables according to their importance arranged by the model, which simultaneously were selected in the LASSO method. Then, a multivariable logistic regression analysis was conducted. Variables with a two-sided *p*-value ≤0.05 and frequently used in routine clinical practice were included in the model along with their odds ratios (ORs), associated 95% confidence intervals (CI), *β*-coefficients, and corresponding *p*-values.

### Machine learning model construction

Regarding machine learning, we used six risk algorithms to develop a predictive model for TS: logistic regression (LR), neural network (NN) (13), random forest (RF) (14), decision tree (DT) (15), Gaussian naïve Bayes (Gaussian NB) (16), and K-nearest neighbor (KNN) (17). LR is basically a classification algorithm that comes under the supervised category. DT is a nonparametric supervised learning algorithm consisting of upside-down trees that make decisions based on the conditions present in the data. RF is a combination of a multitude of decision trees that can be constructed for prediction when facing regression tasks. NN is one of the supervised machine learning methods that simulates the way the human brain processes information. NB is a method based on Bayes theorem mainly used for classification. KNN is a nonparametric classification approach widely used in real-life issues (18–22).

Once the features were inputted, these algorithms enabled predictions regarding important signs for the diagnosis of TS in a sample of patients with TS or BS. R programming software (version 4.1.2) was used to build the predictive models.

### Evaluation and improvement of model performance

The data used in this study were randomly divided into two groups including a training set and a validation set with a ratio of 7:3. Model establishment consists of some unavoidable processes: data preprocessing, training the model with tuned hyperparameters (also called model performance improvement), evaluating the model performance, and testing the model on unknown data. However, previous research studies present an error-prone manipulation, which is reporting the performance estimated in the tuning procedure as model performance, which is somehow biased and overestimated (23). Evaluating the model performance should not be carried on the same datasets used for tuning since this kind of operation would cause biased performance during evaluation. Thus, we adopted a nested resampling strategy (nested cross-validation) to obtain an unbiased score. It used outer and inner loops to separate resampling optimization from model performance evaluation. The model was fitted on the outer training data set using the tuned hyperparameter configuration obtained by inner resampling. Repeated *k*-fold cross-validation (KCV, *k* = 10, *n* = 10, *n* is the number of repeats) was used as the outer resampling strategy, and *k*-fold cross-validation (KCV, *k* = 5) was the inner resampling method to tune the hyperparameters of each model. In the process of KCV, *k*−1 folds of the data were used as the training set and the reserved part of data was used as the testing set to evaluate nine metrics, namely, sensitivity, specificity, accuracy, precision, positive predictive value (PPV), negative predictive value (NPV), F1 score, area under the curve of the receiver operating characteristic curve (AUROC), and the precision–recall curve (AUPRC) iteratively until every fold experienced inner validation. The whole process was repeated 100 times. This was believed to reduce the probability of overfitting and underfitting in a tiny data set and would help to reflect its practical performance.

Ultimately, the values of AUROC and AUPRC from the six models were compared to decide the best performing model. The opted model, logistic regression (LR), was constructed as a scoring system using the entire training data, and it was validated using the validation data set. The ROC and PRC analyses were carried out utilizing the R package: ModelMetrics (version 1.2.2.2) (24).

### Scoring system development and validation

The logistic regression model, selected after the aforementioned individual models were evaluated based on the required criteria, is displayed as a scale system embedded into Excel (Microsoft, USA), which is convenient to use (25). We estimate the discrimination performance of the scale system with AUROC and the calibration curve in the training and validation sets, respectively. At last, decision curve analysis (DCA) was used to examine the clinical efficiency of the model to quantify the benefits and the area under the curve to be appraised (26).

### Statistical analysis

We performed all statistical analyses by using R software 4.1.2. The normality of the data with the *Q*–*Q* plots of all data was assessed. Continuous variables were presented as mean  ± standard deviation (SD) in the case of normal distribution; otherwise, they were presented as median values (quartiles). Student's *t*-test was used to compare two mean values of continuous data considered normally distributed after normality evaluation. Otherwise, the Mann–Whitney *U*-test was performed. Categorical variables were expressed as frequency (percentage). The chi-square test or Fisher's exact test was used to compare two frequencies.

## Results

### Epidemiology of cases enrolled in this study

Regional distributions of patients diagnosed with TS or BS enrolled in this study are shown in Figure 1. For each region, the darker shade represents a higher incidence of disease. As can be seen, in general, the southern part of Xinjiang China, especially the Hotan region, reveals a higher prevalence.

### Patients

A total of 410 patients (*n* = 275 TS patients and *n* = 135 BS patients) were enrolled; 70% of them were included in the training set (*n* = 292), and the remaining patients were included in the validation set (*n* = 118). The differences in all baseline demographic characteristics and predictors, including clinical personation, laboratory tests, and radiology findings between the TS and BS, are given in Table 1. Patients with TS had higher CRP levels, ESR, and proportion of lower pain, while patients with BS showed higher WBC count. In additon, most imaging-related data showed significant differences between patients with TS and BS.

### Feature selection

**Figure 2:**
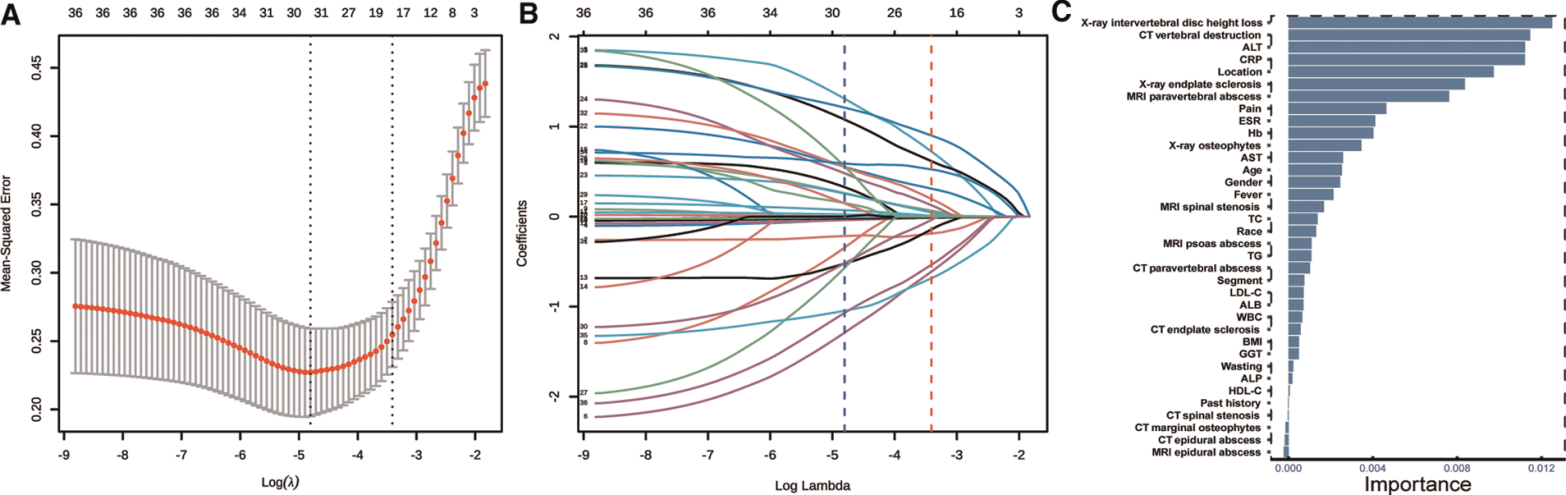
Feature selection. (**A**) Optimal parameter (lambda) selection in the LASSO model using 10-fold cross validation via minimum criteria (the left dotted vertical line) and the 1−SE of the minimum criteria (the right dotted vertical line). (**B**) LASSO coefficient profiles of the 36 features. A coefficient profile plot was produced against the log (lambda) sequence. Nineteen features with nonzero coefficients were selected by the optimal *λ*. (**C**) Features selected using permutation importance *via* random forest ordered by their importance score. LASSO, least absolute shrinkage and selection operator.

Thirty-six variables were reduced to 19 predictors with the LASSO method (Figures 2A,B). The top 10 variables with relative importance score selected by the LASSO method were CRP, ESR, Hb, ALT, pain severity, CT vertebral destruction, x-ray intervertebral disc height loss, x-ray endplate sclerosis, MRI paravertebral abscess, and location (Figure 2C). Multivariate analysis was conducted based on the above results. Predictors associated with the TS patients included pain severity, CRP, x-ray intervertebral disc height loss, x-ray endplate sclerosis, CT vertebral destruction, and MRI paravertebral abscess (Table 2).

**Table 2:** Prediction factors for TS from study population by multiple logistic regression model.

Characteristic	OR	95% CI	*p*-Value
Pain severity			
Moderate	—	—	
Severe	0.37	0.20, 0.66	**<0**.**001**
CRP (mg/L)	1.02	1.01, 1.03	**<0**.**001**
Hb (g/L)	0.97	0.95, 0.99	0.008
ALT (U/L)	0.99	0.98, 1.00	0.045
ESR (mm/h)	0.99	0.97, 1.01	0.2
X-ray endplate sclerosis			
No	—	—	
Yes	0.20	0.11, 0.36	**<0**.**001**
X-ray intervertebral disc height loss			
No	—	—	
Yes	3.31	1.87, 5.98	**<0**.**001**
CT vertebral destruction			
Mild (≤ 1/3)	—	—	
Severe (>1/3)	3.21	1.78, 5.87	**<0**.**001**
MRI paravertebral abscess			
No	—	—	
Yes	3.05	1.72, 5.51	**<0**.**001**
Location			
C	—	—	
C + T	1.92	0.04, 102	0.7
L	4.46	0.89, 26.9	0.079
L + S	3.95	0.69, 26.8	0.13
T	38.6	6.15, 292	**<0**.**001**
T + L	16.5	1.93, 171	0.013

OR, odds ratio; CI, confidence interval; CRP, preoperative C-reactive protein (mg/L); Hb, preoperative hemoglobin (g/L); ESR, preoperative erythrocyte sedimentation rate (mm/h); ALT, preoperative alanine aminotransferase (U/L); C, cervical spine; T, thoracic spine; T + L, thoracolumbar spine; L, lumbar spine; L + S, lumbosacral spine.

### Evaluation of model prediction capability

**Figure 3:**
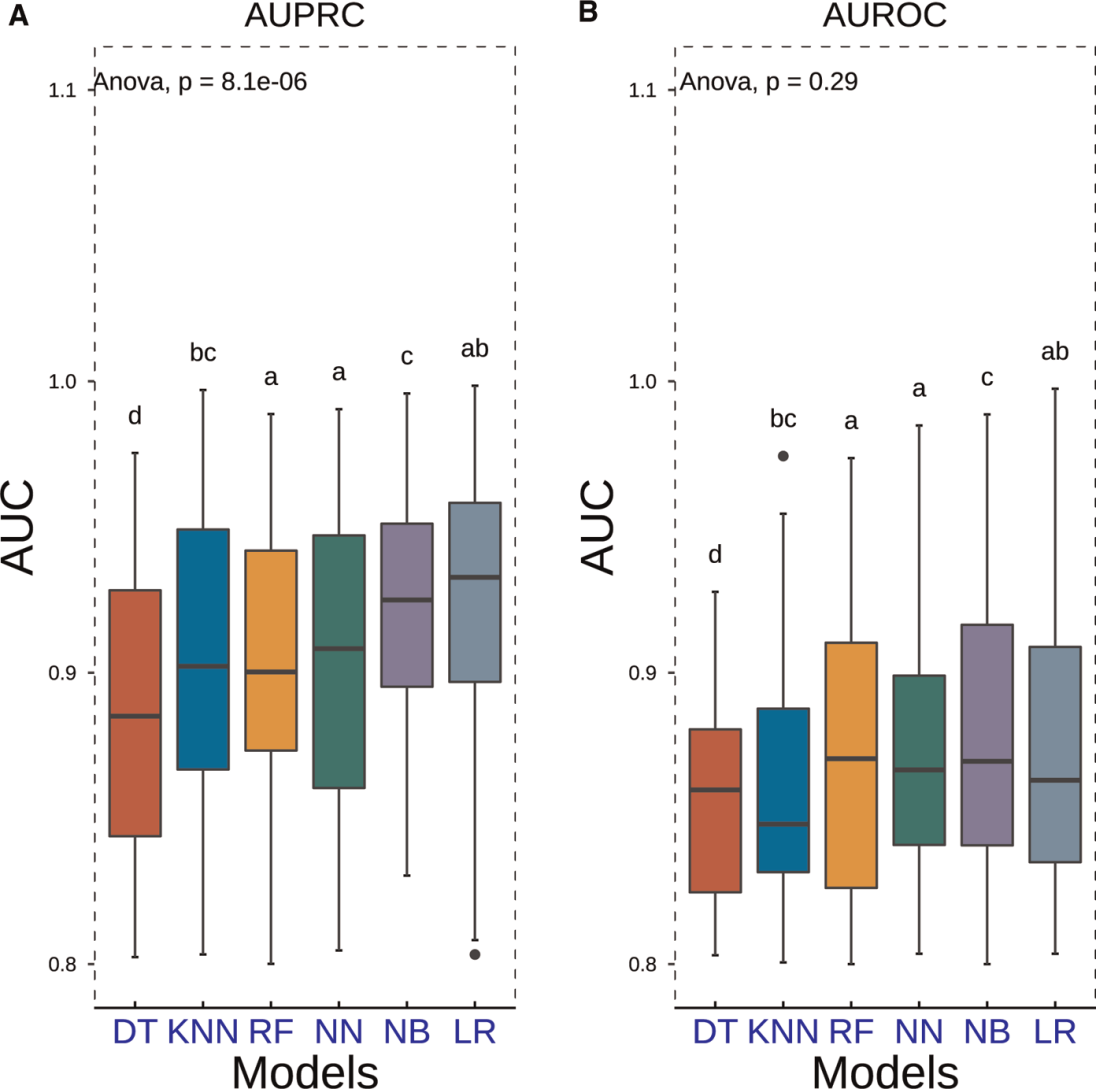
Boxplots of AUPRC and AUROC measurements of model performance using the nested resampling strategy for six different machine learning algorithms. *P-*values were calculated through one-way analysis of variance with Tukey’s *posthoc* test. AUPRC, area under the curve of the receiver operating characteristic curve; AUPRC, area under the curve of the precision–recall curve.

Repeated 10-fold cross-validation was carried out in the outer loop to assess model performance with ROC and PRC analyses. This process was repeated 10 times. We discovered that DT was related to relatively lower AUROC and AUPRC values. However, LR, NN, and NB methods exhibited higher AUROC and AUPRC values (Figure 3). Furthermore, seven popular metrics (sensitivity, specificity, accuracy, precision, F1 score, PPV, and NPV) were also used to assess the performance of these models (Table 3). As LR shows higher specificity than NN and NB and has best accuracy and F1 score, it is the most commonly used algorithm with its convenience displaying high accuracy with lower standard deviance. This indicated that the LR model did possess an outstanding ability to be implemented into clinical decision-making.

**Table 3:** Predictive performance of each model.

Model	Sen.	Spe.	Acc.	Pre.	P.P.V.	N.P.V.	F1
LR	0.88 ± 0.07	0.58 ± 0.18	0.79 ± 0.07	0.82 ± 0.08	0.82 ± 0.08	0.69 ± 0.19	0.85 ± 0.06
NN	0.87 ± 0.08	0.56 ± 0.2	0.77 ± 0.07	0.81 ± 0.09	0.81 ± 0.09	0.68 ± 0.17	0.84 ± 0.06
RF	0.89 ± 0.08	0.56 ± 0.17	0.79 ± 0.08	0.82 ± 0.08	0.82 ± 0.08	0.71 ± 0.18	0.85 ± 0.06
DT	0.86 ± 0.08	0.53 ± 0.19	0.75 ± 0.08	0.8 ± 0.09	0.8 ± 0.09	0.64 ± 0.18	0.82 ± 0.06
NB	0.84 ± 0.09	0.64 ± 0.15	0.77 ± 0.07	0.83 ± 0.08	0.83 ± 0.08	0.66 ± 0.17	0.83 ± 0.06
KNN	0.86 ± 0.06	0.62 ± 0.17	0.78 ± 0.07	0.83 ± 0.08	0.83 ± 0.08	0.67 ± 0.15	0.84 ± 0.05

Sen., sensitivity; Spe., specificity; Acc., accuracy; Pre., precision; P.P.V., positive predictive value; N.P.V., negative predictive value; LR, logistic regression; NN, neural network; RF, random forest; DT, decision tree; NB, naïve Bayes; KNN, K-nearest neighbor.

### Establishment of the scoring system

**Figure 4:**
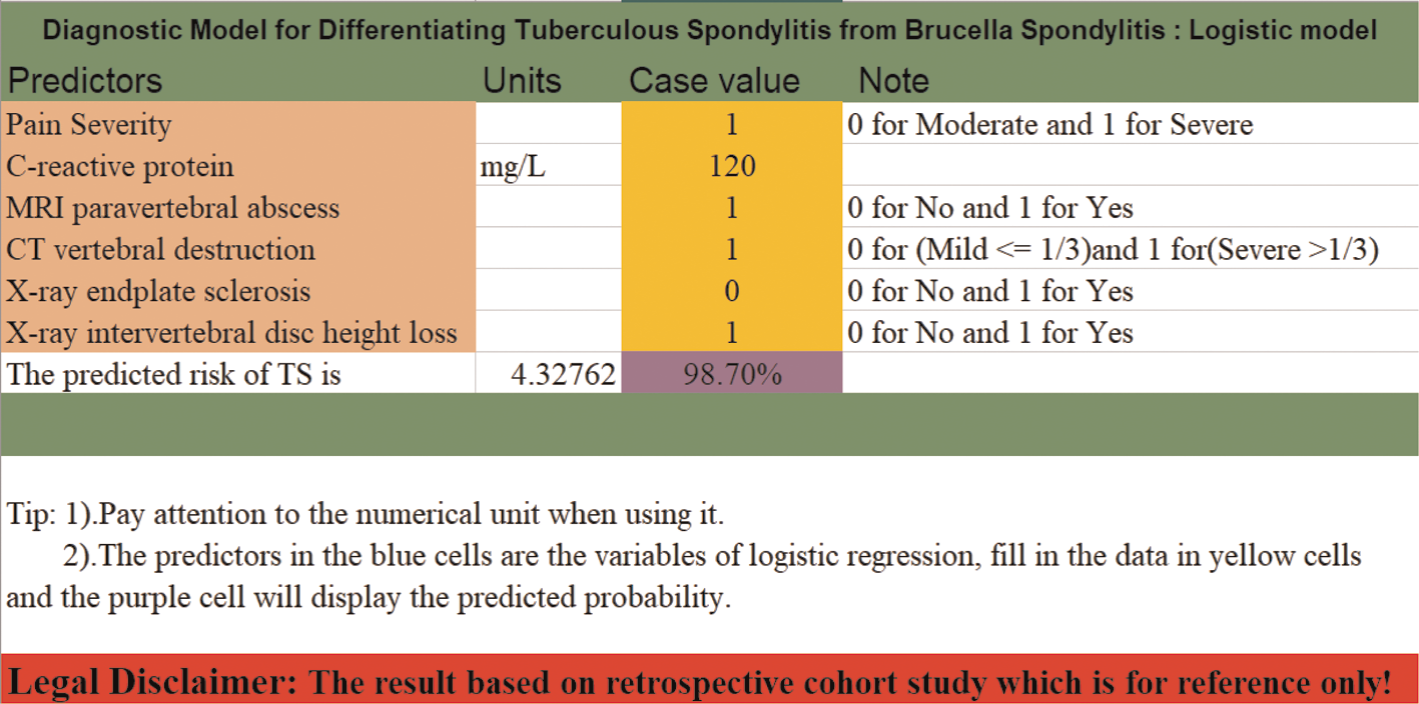
Selected models presented as logistic regression equations in this Excel (USA) document.

Based on the candidate predictors screened on the training set, a scale calculator, which comprised six major features, was developed for predicting the probability of TS. Each factor in the calculator was assigned a unique score in light of the value of the corresponding factor. The sum of all scores computed by rounding up the scores of all predictors can be used to compute the probability of TS (Figure 4). For details, please refer to Table S1.

### Model performance and validation

**Figure 5:**
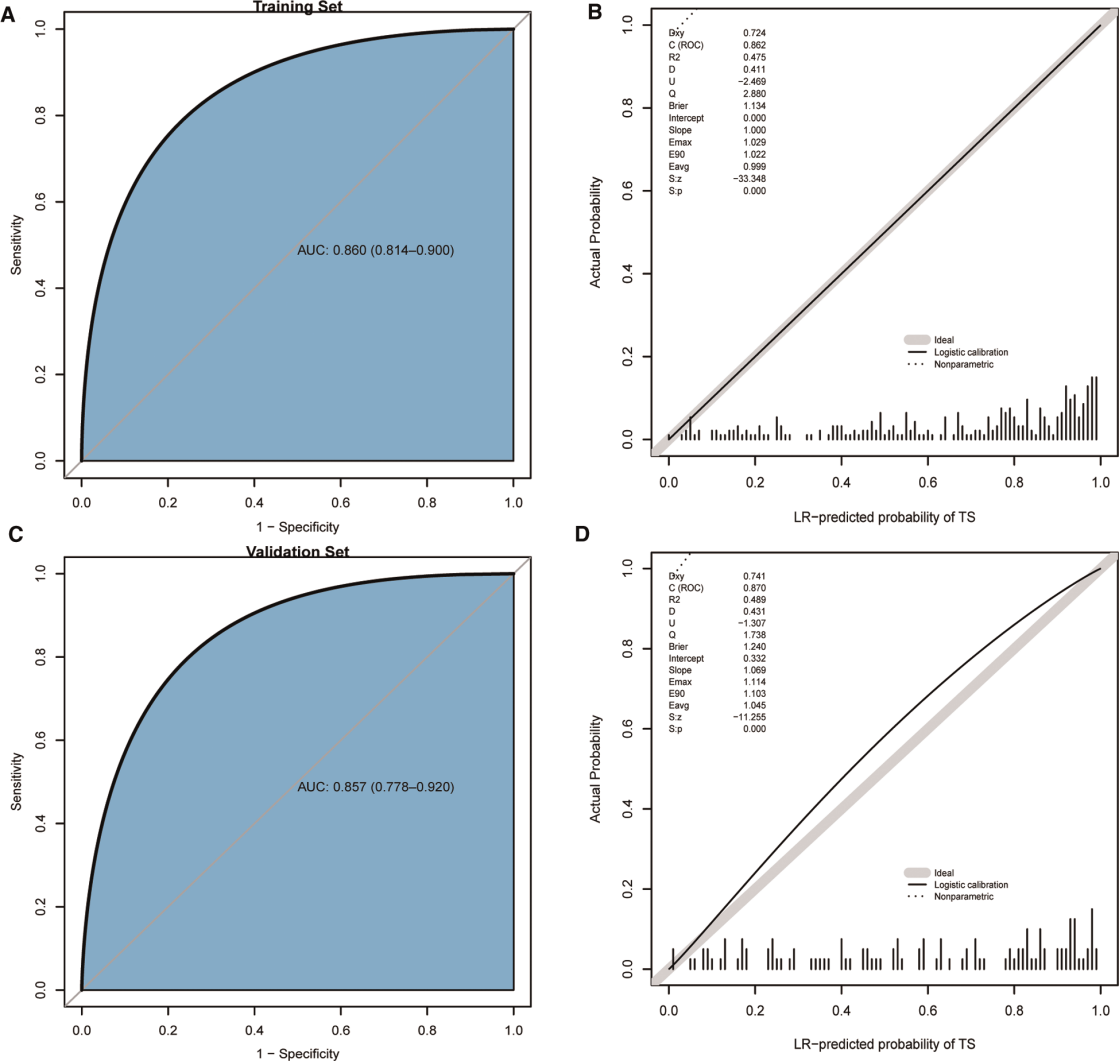
ROC curves and calibration curves of the training set, validation set, and scoring system. (**A**) ROC curve of the training set. (**B**) Calibration curve of the training set. (**C**) ROC curve of the validation set. (**D**) Calibration curve of the validation set. ROC, receiver operating characteristic.

We validated the differentiation capacity of the model in the training set and validation set, respectively. The C-statistics and AUC of the model to predict the diagnosis of TS were 0.860 (95% CI, 0.814–0.900) (Figure 5A) and 0.857 (95% CI, 0.778–0.920) (Figure 5C). The calibration curve showed that the model excellently predicted actual probabilities (Figures 5B,D).

### Clinical efficiency of the model

**Figure 6:**
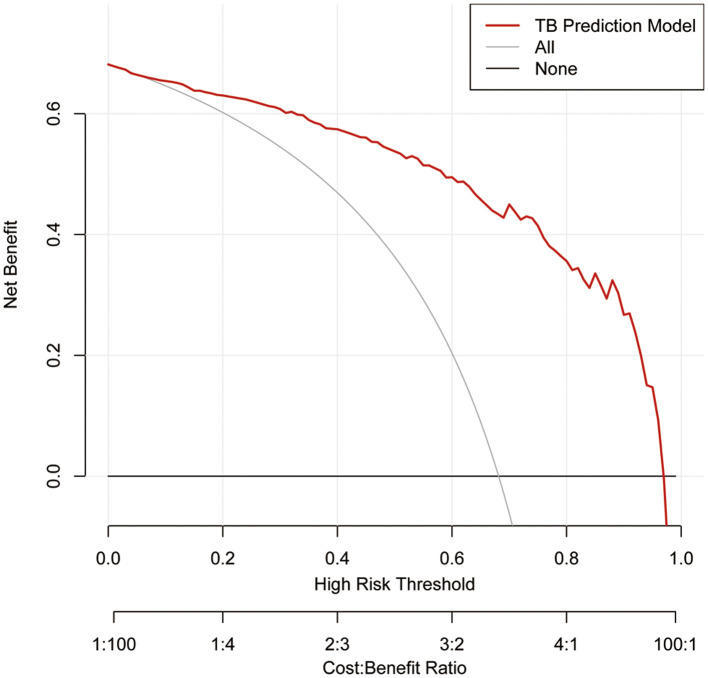
Decision curve analysis for the TS prediction model in the training set. The red line represents the TS predictive model. The thin solid line represents the assumption that all patients are considered to be diagnosed with TS. The thick solid line represents the assumption that no patients suffer from TS. The decision curve analysis indicated that using this TS prediction model could gain net benefit when the threshold probabilities >4%. TS, tuberculous spondylitis.

We implemented DCA to confirm whether it could bring benefit to clinical practice. It can be found that the model had a prominent ability to improve clinical efficiency in predicting TS, as shown in Figure 6.

## Discussion

Machine learning has been widely used in many types of research on diseases. As per our best knowledge, this is the first report on exploiting different machine learning algorithms to develop a diagnostic model with noninvasive clinical indices to differentiate between TS and BS. ML approaches vary their performance depending on various hyperparameters, which play a significant role in decision-making. Finding a set of configurations of hyperparameters is called tuning. It is realized that performance evaluation and tuning are strongly correlated. The nested resampling method we implemented in this research could combined these two procedures to minimize the bias occurring in the whole process. Moreover, the opted model has been visualized as a calculator embedded into an Excel document to encourage further study of its clinical utility. All distinctive predictors selected in the prediction model were basic clinical appearance, laboratory tests, and different imaging data, allowing for routine accessibility in clinical practice. The results displayed that our model possessed excellent discrimination and calibration capacity in two data sets, with AUC values of 0.860 in the training set and 0.857 in the validation set. However, we can find from the above results that the model has the likelihood of misclassification. We assume that this is because of the instability of data. In addition, it somehow depends on the interpretation of the radiologist evaluating the image of patients because the five predictors are related to radiological manifestations.

Both tuberculosis and brucellosis are systemic diseases and remain to be considered public health issues, especially in developing countries, showing higher incidence in the northwest part of China than the other parts of China (27). TS has been mainly discovered in less developed regions because of low income and hygienic status (28). Xinjiang has the second highest incidence of human brucellosis, according to data from the China Public Health Data Center, where patients are mainly pastoralists and veterinarians (29). Previous studies have shown human brucellosis is associated with contact with animals and consumption of uncooked milk and products from goat and sheep (30–32). In addition, there are other factors also connected to brucellosis like high temperatures, air pollution, wind speed, etc. (33). However, the aforementioned factors can be found in Southern Xinjiang, China. Our statistical results based on the patients enrolled in this study displayed that the southern part of Xinjiang, China shows a higher incidence than the northern part, which agrees well with previous research studies. The clinical diagnosis of spinal tuberculosis usually comprises clinical manifestations, laboratory studies, and imaging data (34). The gold standard for diagnosing spinal TB or BS is bacterial isolation (culture) from blood, bone marrow, or tissues (35, 36). Nevertheless, confined to the low positive rate of mycobacteria culture or isolation, diagnosis commonly incorporates clinical symptoms, physical examinations, radiographic findings, tissue a microbiological culture, polymerase chain reaction (PCR), and gene detection (37). Due to the resemblance in the clinical manifestation laboratory tests and imaging findings, many patients may be misdiagnosed during the primary phase of the sickness due to delays from insufficient knowledge (38). Early recognition and effective cure are critical in preventing devastating complications (39). Thus, it is urgent to investigate the related features, develop a convenient and sensitive prediction model, and help primary health care clinicians in less developed areas.

In this article, we select six predictors strongly associated with TS, including pain severity, CRP, x-ray intervertebral disc height loss, x-ray endplate, CT vertebral destruction, and MRI paravertebral abscess. To minimize the heterogeneity of the model to differentiate TS from BS, we chose to acquire features based on the first blood test. We believe that this measure can reduce heterogeneity and boost the model performance.

Patient complaints in TS or BS may initially be effortful to discriminate because of the nature of the illness. Patients with BS often report moderate fever, sweating, malaise, back pain (local pain), and anorexia, whereas patients with TS report back pain, evening pyrexia, generalized body ache, fatigue, body weight loss, neurological abnormalities, and night sweats. Unfortunately, one or more of these symptoms are shown in merely 20%–38% of patients with skeletal tuberculosis (40, 41). Back pain is considered the most frequent complaint of TS. It can be axial pain or radicular pain, which is believed to be the result of the damage to the anterior spinal bodies and mass effect by cold abscess or instability of the spine, nerve root compression, and vertebral body collapse (41, 42). In clinical practice, pain severity showed variance between TS and BS, and the latter can be found with severe pain degrees the former, which is concordant with previous findings. The result of multivariate logistic regression also proved that point (OR: 0.37, 95% CI, 0.20–0.66, *p* < 0.001). Fever types of the two diseases also show differences in that brucellosis appears to be a moderate (≥38.5°C) fever, while tuberculosis is low (<38.5°C) fever with sweats (*p* < 0.001). However, it was not included in our model. Given the wide range among the patients, their age, gender, and ethnicity, to some degree, may affect the result. However, gender shows a great difference between TS and BS, which might be the result of sampling bias. None of these were selected as predictors in ML models because the training set cannot be represented with a small number of samples. Thus, we maintained that there were no significant differences in demographic characteristics, including ethnicity, gender, history of weight loss, history of tuberculosis in other solid organs, and age, between the BS and TS patients after the scientific and precise analysis of our data, which is in line with previous studies (43).

Clinical laboratory tests, such as WBC count, ESR, and CRP level, which are all nonspecific in showing infectious processes and linked to spondylitis in the majority of cases, are a significant part of clinical diagnoses (40, 42, 44, 45). It can be easily found from our result that CRP levels were higher in TS patients than those in BS patients (*p *< 0.001), which was similar to the results reported in previous studies (46–48). At the same time, contrary to the findings, we did not find a significant difference in WBC count and ESR between patients with TS and BS.

Radiological findings are the keystone of the diagnostic process (49). Plain radiography is usually examined first in patients suspected to have TS or BS, and plain radiography images may exhibit no positive result at the early stage of the disease (50). CT has high sensitivity for early diagnosis. In addition, the identification of the extent of the inflammatory process can also be evaluated in time. Moreover, CT has unreplaceable merits of better visualization of the bony details of irregular lytic lesions, sclerosis, disc collapse, and damage to vertebral circumference (51, 52). Previous findings suggest that the diagnosis and differential diagnosis based on MRI of spondylitis patients was qualitative (53, 54). TS and BS are the results of *M. tuberculosis* and *B. melitensis* infections, respectively, which can cause vertebral edema and abscesses, which is reflected by increased T2 values. The lesion level and segments of spinal disease are known to vary according to its etiology. It has been observed that thoracic involvement and multifocal involvement were generally associated with TS (55, 56), a finding consistent with our result. Previous studies have demonstrated that paravertebral abscess, severe bone destruction, and intervertebral disc height loss were suggestive of TS, while local bone damage and confined paravertebral involvements were suggestive of BS, which can be proved by our results (57). In addition to that, endplate sclerosis and osteophytes are more common in BS than in TS, while disc height loss is more frequent in TS, which is in agreement with previous studies (37, 58, 59).

A previous study indicates no sign of predicting the benefit of ML over LR for clinical prediction models (60). The LR model showed good performance with AUROC, AUPRC, and specificity and no significant difference when compared to SVM and NB. Thus, we selected the logistic regression model to differentiate TS from BS. Previous research studies have largely used nomograms exhibiting predictive models. It is not precise enough and somewhat rough to use, and some factors in this model cannot be computed directly, so a scaling system is chosen to visualize the model (25).

## Limitations

There are several limitations of this research. First, this analysis was based on data acquired from electronic medical records in a single center, and it would be more convincing to use multicenter clinical data. Second, it was hard to determine the phase of disease in this series. In addition, as a retrospective design, the research has a few innate demerits compared to a prospective study. What is more, further prospective studies to validate its efficacy with a larger sample size are still needed.

## Conclusions

The model established in this research revealed better discrimination and calibration capability, and internal cross-validation disclosed that this model can still maintain stability when facing diverse tasks. Then, this model was visualized by a calculator that can quickly identify individuals at risk of TS and help physicians in primary health care in less developed areas with a higher incidence of TS or BS in time.

## Data Availability

The original contributions presented in the study are included in the article/**Supplementary Material**; further inquiries can be directed to the corresponding author.
